# 铁死亡在非小细胞肺癌中的作用及中药干预进展

**DOI:** 10.3779/j.issn.1009-3419.2024.101.06

**Published:** 2024-03-20

**Authors:** Xiaoqi GUO, Tianqi WANG, Jinchan XIA, Huahui ZENG, Wenbo SHI

**Affiliations:** ^1^450046 郑州，河南中医药大学医学院; ^1^Medical College, Henan University of Traditional Chinese Medicine, Zhengzhou 450046, China; ^2^450046 郑州，河南中医药科学院; ^2^Academy of Chinese Medicine, Henan University of Traditional Chinese Medicine, Zhengzhou 450046, China

**Keywords:** 肺肿瘤, 铁死亡, 中药, Lung neoplasms, Ferroptosis, Traditional Chinese medicine

## Abstract

非小细胞肺癌（non-small cell lung cancer, NSCLC）是全球发病率和死亡率较高的恶性肿瘤之一。铁死亡是铁依赖性活性氧（reactive oxygen species, ROS）异常堆积导致脂质过氧化而引起的新型细胞程序性死亡方式，涉及铁代谢、脂质代谢、氧自由基反应与脂质过氧化之间的平衡。近年来研究发现铁死亡与NSCLC发生发展密切相关。由于NSCLC治疗过程中化疗耐药及放疗抵抗等的出现，迫切需要开发新的有效药物和治疗策略，中药具有靶点多、副作用小等特点，在防治NSCLC方面有独特的优势。本文综述了铁死亡在NSCLC中的作用机制，探讨中药活性成分、单味药及中药复方通过铁死亡干预NSCLC的研究现状，以期为铁死亡通路研究及中药靶向铁死亡防治NSCLC提供新的理论依据。

## Abstract (en)

Non-small cell lung cancer (NSCLC) is one of the malignant tumors with high morbidity and mortality worldwide. Ferroptosis is a new type of programmed cell death caused by abnormal accumulation of iron-dependent reactive oxygen species (ROS) leading to lipid peroxidation. It involves the balance between iron metabolism, lipid metabolism, oxygen free radical reaction and lipid peroxidation. Recent studies have found that ferroptosis is closely related to the occurrence and development of NSCLC. Due to the emergence of chemotherapy resistance and radiotherapy resistance in the treatment of NSCLC, there is an urgent need to develop new effective drugs and treatment strategies. Traditional Chinese medicine has unique advantages in the prevention and treatment of NSCLC due to its multi-targets and minimal side effects. In this review, we summarize the mechanism of ferroptosis in NSCLC, and discuss the research status of active ingredients of traditional Chinese medicine, single-herb traditional Chinese medicine and Chinese herbal compounds in the intervention of NSCLC through ferroptosis, in order to provide a new theoretical basis for the research of ferroptosis pathway and the prevention and treatment of NSCLC by targeted ferroptosis of traditional Chinese medicine.

肺癌是全球高发病率和高死亡率的恶性肿瘤之一，在我国每年超过70万人确诊为肺癌，约占所有新发癌症病例的16.7%，死亡率占所有癌症死亡人数的24.4%，严重危害人类健康^[1]^。其中非小细胞肺癌（non-small cell lung cancer, NSCLC）是肺癌的主要亚型，约占所有肺癌病例的85%。目前NSCLC的治疗选择包括手术和辅助治疗（如放疗、化疗、靶向治疗和免疫疗法）。其中手术治疗只适用于肺癌早期，同时随着肺癌细胞对辅助治疗产生不同程度的抵抗性和耐药性，导致肺癌的治疗往往不尽如人意^[2]^，因此探索NSCLC新的干预靶点及治疗药物迫在眉睫。

铁死亡是一种铁依赖性活性氧（reactive oxygen species, ROS）异常堆积导致膜质过氧化而引起的一种新型细胞程序性死亡（programmed cell death, PCD）方式^[3]^。与其他PCD不同的是，铁死亡主要特征包括铁离子异常累积、脂质过氧化作用增加、ROS增加、线粒体电位下降或消失、线粒体嵴减少、染色质浓缩以及一些特征性基因（TfR1、SLC7A11、GPX4等）表达改变^[4]^（表1）。铁是细胞生长必需元素，与正常细胞相比，癌细胞快速增殖的特点使其对铁有更强的依赖性^[5]^，而铁代谢失衡又会进一步增加患癌的风险，因此通过促进肿瘤细胞铁死亡抑制NSCLC的生长、增殖、转移、上皮间充质转化，逆转其耐药性，可能会成为肺癌治疗所面临问题的一个突破口^[6]^。中药在治疗肺癌方面具有疗效确切、副作用小的特点，但作用机制尚不明确，既往研究发现一些中药及活性成分可以通过诱导NSCLC细胞铁死亡进而抑制肿瘤的生长、增殖、转移、上皮间充质转化，并逆转耐药性^[7]^。因此，本文综述了铁死亡在NSCLC中的作用及中药干预作用的研究进展，旨在为铁死亡通路研究及中药靶向铁死亡防治NSCLC提供理论基础。

**表1:** 细胞程序性死亡方式的区别

Type of PCD	Morphological characteristics of cells	Indicators of detection
Apoptosis	The cytoplasm was dehydrated and concentrated, the cell membrane was vacuolated, the cell volume was reduced and pyknotic, the endoplasmic reticulum was dilated and vesicular and fused with the cell membrane, the nucleolus was cleaved, and apoptotic bodies were formed	The levels of MMP, PS and apoptosis-related proteins such as Caspase-3, PARP, etc.
Necroptosis	Cell size increases, organelles swell, membranes perforate, and finally cells disintegrate	The levels of necroptosis-related proteins such as RIPK3, RIPK1, and MLKL, etc.
Autophagy	The Golgi apparatus, endoplasmic reticulum and other organelles were swollen, the cytoplasm was amorphous, the nucleus was fragmented and pyknotic, and a large number of phagocytic vacuoles may occur	The number of autophagosomes and the levels of autophagy related proteins such as ATG5, ATG7, BeclinI, LC3, P62, etc.
Pyroptosis	The cell swells, and before the cell ruptures, a bulge forms on the cell, after which pores form in the cell membrane, causing the cell membrane to lose its integrity, releasing contents, causing inflammation, nuclear pyknosis, and DNA fragmentation	The levels of LDH, IL-1β, IL-18 and pyroptosis-related proteins Caspase-1, Gasdermin D, Caspase-4, etc.
Ferroptosis	Mitochondrial cristae decreased (disappeared), mitochondrial outer membrane rupture and shrinkage, the color of mitochondria was dark	The levels of Intracellular Fe^2+^, ROS, GSH, GPX4，lipid peroxides MDA and LPO and ferroptosis-related proteins such as TfR1, SLC7A11, GPX4, ACSL4, LPCAT3, FTH1, etc.

MMP: mitochondrial membrane potential; PS: phosphatidylserine; PARP: poly ADP-ribose polymerase; RIPK3: receptor-interacting serine-threonine kinase 3; RIPK1: receptor interacting serine/threonine kinase 1; MLKL: mixed lineage kinase domain-like; ATG5/7: autophagy protein 5/7; LDH: lactate dehydrogenase; GSH: glutathione; GPX4: glutathione peroxidase 4; LPO: lipid peroxidation; ACSL4: acyl-CoA synthetase long-chain family member 4; LPCAT3: lysophosphatidylcholine acyltransferase 3; TfR1: transferrin receptor 1; SLC7A11: solute carrier family 7 member 11; FTH1: ferritin heavy chain 1; PCD: programmed cell death; IL: interleukin.

## 1 铁死亡与NSCLC

### 1.1 铁代谢紊乱

铁是人体所需的基本微量元素之一，细胞外游离的Fe^3+^与转铁蛋白（transferrin, TF）结合形成铁蛋白复合物，该复合物与细胞膜上的转铁蛋白受体1（transferrin receptor 1, TfR1）结合后进入细胞^[8]^。进入细胞后，Fe^3+^被转送至内涵体（endosomes）中，在STEAP3（six transmembrane epithelial antigen of prostate 3）作用下还原成Fe^2+^，随后被DMT1（divalent metal transporter 1）释放到细胞质中形成不稳定铁池，过量的Fe^2+^可以储存在铁蛋白（ferritin）或被铁转运蛋白（ferroportin, FPN/SLC40A1）转移到细胞外，确保细胞内铁代谢平衡^[9]^。当NCOA4（nuclear receptor coactivator 4）诱导的铁蛋白降解增加或FPN受到抑制时会导致Fe^2+^过载^[10,11]^，Fe^2+^可以与过氧化氢（H_2_O_2_）发生芬顿（Fenton）反应（Fe^2+^+H_2_O_2_→Fe^3+^+•OH+OH^−^）产生大量ROS，进而诱导膜质过氧化，最终导致细胞铁死亡的发生^[12]^。而铁螯合剂（deferoxamine, DFO）和铁死亡抑制剂（ferrostatin-1, Fer-1）可以与细胞内铁离子结合抑制铁死亡^[13]^。

铁代谢紊乱在肿瘤细胞中普遍存在，在NSCLC中铁代谢紊乱主要表现为铁输入增加和输出抑制，从而造成铁蓄积，这导致细胞对铁死亡更敏感。通过构建含铁离子的纳米粒，递送Fe^2+^至NSCLC细胞中，激活Fenton反应诱导铁死亡可以抑制细胞的生长、增殖、迁移并逆转其化疗耐药^[14,15]^。细胞色素P450还原酶（cytochrome P450 reductase, POR）是一种微粒体（microsomes）内的膜结合蛋白，POR通过上调多不饱和脂肪酸的脂质过氧化促进铁死亡发生，铁离子可以作为电子接收剂或者氧化剂参与反应^[16]^，POR与细胞色素B5还原酶（cytochrome B5 reductase, CYB5R1）还可以将电子从NAD（P）H转移到氧分子中生成H_2_O_2_，过氧化氢通过Fenton反应产生活性羟自由基，进而破坏细胞膜的完整性^[17]^。Yap（Yes-associated protein）是Hippo肿瘤抑制通路中的关键转录因子，对NSCLC的生长、转移和耐药具有显著的促进作用^[18,19]^。研究^[20]^表明，通过抑制Yap可以减少铁蛋白重链（ferritin heavy chain, FTH）和铁蛋白轻链（ferritin light chain, FTL）的合成来控制细胞内的铁水平，进而增强NSCLC铁死亡的敏感性。miR-302a-3p通过与FPN的3'-UTR直接结合，诱导脂质过氧化、铁过载和铁死亡，从而抑制NSCLC细胞的生长和集落形成，并增强其对顺铂和紫杉醇化疗敏感性^[21]^。长非编码RNA（long non-coding RNA, lncRNA）H19作为竞争性内源性RNA与miR-19b-3p结合，从而增强FTH的转录活性，促进铁死亡^[22]^。海洋生物活性碱（fascaplysin）通过增加Fe^2+^和ROS水平诱导铁死亡，并提高抗程序性死亡受体（programmed cell death 1, PD-1）免疫治疗的疗效^[23]^。

### 1.2 脂质过氧化

细胞膜主要是由脂类、蛋白质、糖组成，其完整性对维持细胞正常功能至关重要^[24]^。当细胞中ROS过多时会与膜中磷脂的多不饱和脂肪酸（polyunsaturated fatty acid, PUFA）侧链结合，发生脂质过氧化反应生成大量脂质过氧化产物（lipid peroxide, LPO），破坏细胞膜的流动性和通透性，导致细胞死亡。酰基辅酶A长链合成酶4（acyl-CoA synthetase long-chain family member 4, ACSL4）可以较好地连接长链PUFA，包括花生四烯酸（arachidonic acid, AA）和肾上腺素酰基（adrenoyl, AdA），生成酰基CoA衍生物（CoA derivatives）^[25]^，然后溶血磷脂酰胆碱酰基转移酶3（lysophosphatidylcholine acyltransferase 3, LPCAT3）将这些衍生物酯化成磷脂酰乙醇胺（arachidonic acid phosphatidylethanolamine, AdA-PE; adrenic acid phosphatidylethanolamine, AA-PE）^[26]^，最后，AA-PE和AdA-PE被脂氧合酶15（arachidonic acid lipoxygenase 15, ALOX15）氧化生成LPO。ALOX15是一种含铁酶，磷酸化酶激酶G2（phosphorylating enzyme kinase G2, PHKG2）通过调节细胞内的铁离子浓度，影响ALOX15对PUFA的氧化反应^[27]^。

LncRNA NEAT1可以靶向抑制ACSL4的蛋白表达，从而降低NSCLC细胞对铁死亡的敏感性^[28]^。LncRNA ASMTL‐AS1通过招募U2AF2（U2 small nuclear RNA auxiliary factor 2, U2AF2）来稳定SAT1（spermine N1‐acetyltransferase 1, SAT1），SAT1正向调节ALOX15表达诱导NSCLC铁死亡发挥抗癌作用^[29]^。

### 1.3 抗氧化系统失调

#### 1.3.1 胱氨酸/谷氨酸反向转运体（cystine/glutamate antiporter, System Xc^-^）途径

为了抵御膜质过氧化的损害，细胞内存在一些重要的抗氧化系统，首先是经典的谷胱甘肽过氧化物酶4（glutathione peroxidase 4, GPX4）依赖通路，该通路主要通过System Xc^-^发挥作用，该系统位于细胞膜上，由轻链SLC7A11（xCT）和重链SLC3A2（4F2hc）组成，按照1:1的比例输入胱氨酸（cystine）和输出谷氨酸（glutamate）^[30]^，胱氨酸进入细胞后进一步生成为谷胱甘肽（glutathione, GSH），而GPX4以GSH为底物将LPO还原为对细胞膜无害的脂醇，抑制铁死亡发生^[31]^。当System Xc^-^受到抑制时，GSH转化不足，导致细胞抗氧化能力减弱诱发铁死亡^[32]^。

SRY-box转录因子2（SRY-box 2, SOX2）可以与SLC7A11启动子结合促进其表达，增加细胞内的胱氨酸，SOX2高表达使NSCLC细胞对铁死亡抵抗力更强^[33]^。RNA结合基序单链相互作用蛋白1（RNA binding motif single stranded interacting protein 1, RBMS1）通过与翻译起始因子EIF3D（eukaryotic translation initiation factor 3 subunit D）相互作用，桥接SLC7A11的3'-UTR和5'-UTR，促进SLC7A11的翻译抑制铁死亡发生，且RBMS1高表达与NSCLC患者生存率降低有关^[34]^。甲基转移酶样蛋白3（methyltransferase-like 3, METTL3）介导SLC7A11 mRNA的m6A（N6-methyladenosine）修饰，并通过招募m6A阅读器YTHDF1（YTH domain family 1）稳定SLC7A11 mRNA并促进其翻译，从而促进NSCLC细胞增殖并抑制细胞铁死亡^[35]^。含有TCP1亚基3的伴侣蛋白（chaperone protein containing TCP1 subunit 3, CCT3）促进细胞周期进程并抑制SLC7A11介导的细胞铁死亡，且CCT3高表达与肺腺癌患者的不良预后有关^[36]^。在肺腺癌中，甲基腺苷RNA结合蛋白C2（YTH N6-methyladenosine RNA binding protein C2, YTHDC2）不仅可以抑制SLC7A11，而且以m6A依赖的方式破坏同源框蛋白A13（homo box A13, HOXA13）mRNA的稳定性，HOXA13作为转录刺激因子还可以下调SLC3A2的表达进而促进铁死亡发生^[37]^。miR-27a-3p可以与SLC7A11的3'-UTR直接结合，抑制SLC7A11的表达^[38]^。环状RNA FOXP1（CircFOXP1）在NSCLC患者血清中表达量显著增高^[39]^，研究^[40]^发现Circ FOXP1可以与miR-520a-5p结合，从而上调SLC7A11的表达，抑制铁死亡并促进肿瘤细胞增长。环状RNA P4HB（CircP4HB）通过调节miR-1184，进而上调SLC7A11的表达来抑制铁死亡^[41]^。铁死亡诱导剂PRLX93936能够抑制System Xc^-^表达，降低GSH和GPX4的活性，增强顺铂治疗NSCLC的效果^[42]^。p53是一种重要抑癌基因，其可以促进泛素特异性蛋白酶7（ubiquitin-specific protease 7, USP7）的核转位，从而抑制SLC7A11的表达，但50%-70%的NSCLC患者伴随p53基因突变，无法发挥正常功能^[43]^；P53还可以通过增强SAT1和谷氨酰胺酶2（glutaminase 2, GLS2）的表达，抑制胱氨酸的摄取，从而增强了NSCLC细胞对铁死亡的敏感性^[44]^。左旋丁哌卡因、Erastin和含有P53激活肽纳米粒子都可以在体内外均可激活P53信号通路，从而抑制NSCLC的发生发展^[45⇓-47]^。金霉素和奥拉帕尼联用时通过ROS介导的细胞铁死亡途径来杀死p53基因突变的NSCLC细胞，效果优于单用奥拉帕尼^[48]^。LncRNA P53RRA可以与G3BP1（Ras GTPase-activating protein-binding protein 1）结合激活P53通路，并增加NSCLC细胞内Fe^2+^和ROS浓度诱导铁死亡^[49]^。

#### 1.3.2 谷胱甘肽过氧化物酶4（glutathione peroxidase 4, GPX4）通路

GPX4是细胞中的一种抗氧化酶，在NSCLC生长、增殖、上皮间充质转化、耐药中发挥重要作用，GPX4高表达与NSCLC患者较差的临床预后相关^[50,51]^。GPX4能够将细胞内的脂质过氧化物还原成无害的脂醇，抑制肿瘤细胞的铁死亡^[52]^。Notch家族成员Notch3（Notch homolog 3）的敲低可以增加细胞内ROS水平，并伴随GPX4和过氧化物还原蛋白6（peroxiredoxin 6, PRDX6）的表达水平下调，诱导脂质过氧化反应触发NSCLC细胞铁死亡^[53]^。肺腺癌细胞株SW900中的STYK1（serine-threonine tyrosine kinase 1）基因的过表达上调了GPX4的表达，促进细胞增殖并抑制铁死亡^[54]^。环状RNA DTL（Circ-DTL）通过上调Circ-DTL/miR-1287-5p/GPX4轴抑制铁死亡和凋亡发挥致癌作用，沉默Circ-DTL可促进NSCLC细胞对化疗药物的敏感性并抑制体内肿瘤的生长^[55]^。异丙酚能上调miR-744-5p和miR-615-3p以抑制GPX4的转录，通过增强NSCLC铁死亡敏感性并逆转顺铂耐药性^[56]^。miR-324-3p可以靶向GPX4基因并抑制其表达，通过上调miR-324-3p的表达，从而诱导NSCLC细胞发生铁死亡，并逆转NSCLC细胞顺铂耐药^[57]^。他汀类药物通过降低细胞内GPX4的含量诱导细胞铁死亡，并在转录水平抑制程序性死亡配体1（programmed cell death-ligand 1, PD-L1）表达增强免疫治疗的疗效^[58]^。

#### 1.3.3 其他调控机制

铁死亡抑制蛋白1（ferroptosis suppressiprotein 1, FSP1）可以将细胞质膜上的辅酶Q10（co-enzyme Q10, CoQ10）还原为泛醌醇（coenzyme QH2, CoQH2），醌氧化还原酶1[NAD(P)H dehydrogenase quinone 1, NQO1]在此过程中发挥关键作用，并将维生素K还原为氢醌，CoQH2和氢醌发挥捕获自由基的作用，使细胞免受铁死亡损伤，这一通路也被称为非经典的GPX4通路^[59]^。miR-4443通过靶向METLL3并以m6A方式增加FSP1的表达从而抑制铁死亡发生，增强NSCLC细胞顺铂耐药性。线粒体二氢乳清酸脱氢酶（dihydroorotate dehydrogenase, DHODH）能将CoQ10还原为CoQH2，抑制线粒体膜的脂质过氧化和铁死亡^[60]^。NQO1在NSCLC中高表达，通过降低NQO1的含量，可以显著抑制NSCLC细胞的生长和扩散并可以降低顺铂的耐药性^[61]^。四氢生物喋呤（tetrahydrobiopterin, BH4）是GPX4受到抑制后的重要抗氧化途径，BH4能够捕获自由基避免脂质过氧化，进而抑制铁死亡发生^[62]^。有研究^[63,64]^表明，铁死亡的发生依赖于自噬，自噬调节因子可以影响铁死亡的发生，特别是NCOA4介导的铁蛋白吞噬、自噬调节蛋白Beclin 1与SLC7A11直接结合都通过自噬调节铁死亡的发生。

核因子E2相关因子2（nuclear factor erythroid 2-related factor 2, Nrf2）是一种重要的抗氧化转录因子，其高表达也是NSCLC耐药的主要原因之一^[65]^。正常情况下，Nrf2与KEAP1（Kelch-like ECH-associated protein 1）结合，KEAP1作为泛素连接酶的接头亚基，指导Nrf2在蛋白酶体降解。在氧化应激条件下，Nrf2会释放并转位到细胞核中，激活血红素加氧酶-1（heme oxygenase-1, HO-1），HO-1通过降解血红素生成一系列的具有抗氧化、抗炎作用的产物，对抗超氧化物和自由基对细胞的损害^[66]^。miR-6077通过调控KEAP1-Nrf2-SLC7A11/NQO1轴抑制铁死亡，保护NSCLC细胞免受顺铂联合培美曲塞诱导的细胞死亡^[67]^。System Xc^-^的活性和表达水平也受到Nrf2的调控，Nrf2的激活可以进一步增加System Xc^-^的表达，同时Nrf2是谷氨酸半胱氨酸连接酶（glutamate-cysteine ligase, GCLC）、谷胱甘肽合成酶（glutathione synthetase, GSS）等的关键转录因子，通过促进GSH的合成抑制铁死亡的发生^[68]^。异位表达lncRNA MT1DP可以稳定miR-365a-3p并下调Nrf2，使NSCLC细胞GSH水平下降增强铁死亡敏感性^[69]^。当Nrf2被E3泛素连接酶（E3 ubiquitin ligase, MIB1）降解时，NSCLC细胞的抗氧化能力减弱，肺癌细胞对铁死亡诱导剂的敏感性增强^[70]^。泛素特异性加工蛋白酶11（ubiquitin-specific-processing protease 11, USP11）在NSCLC患者中高表达，通过去泛素化保持Nrf2稳定性，USP11的耗竭有助于促进铁死亡并抑制细胞增殖^[71]^。索拉非尼与Erastin联用时通过抑制Nrf2/System Xc^-^通路，降低NSCLC细胞内抗氧化防御系统的活性并减少半胱氨酸的摄取，从而导致NSCLC细胞内过氧化脂质的积累和铁死亡的发生^[72]^。综上，抑制Nrf2或促进其降解可能有利于NSCLC铁死亡发生。

## 2 中药调控NSCLC细胞铁死亡的研究进展

中医认为，肺癌是机体正气内虚，邪毒侵肺，气滞血瘀，痰浊内聚，于是邪气瘀毒交结，日久形成肺部积块，属于“肺积”“息贲”等的范畴，扶正祛邪、标本兼治是中医治疗肺癌的基本原则。肺癌早期以邪实为主，邪气盛则实，治当行气活血、清热解毒、化痰软坚等，所谓邪去则正安；晚期以正虚为主，精气夺则虚，治当益气养阴、补气生血、补阴温阳等，即正气存内，邪不可干^[73]^。目前NSCLC治疗过程中出现的药物耐药性和毒副作用的缺点，严重影响了疗效和患者的生存质量，而中药在抗NSCLC方面展现出独特优势：耐药性低、毒副作用小、综合调节机制等，但是其物质基础和作用机制尚不完全明确^[74]^。随着现代生物学技术的不断发展，探究中药抗NSCLC的分子调控机制成为研究热点，而铁死亡理论的提出部分解释了中药抗NSCLC的机制，并为中药的开发和研究提供了重要的指导。本文汇总了可以诱导NSCLC细胞发生铁死亡的中药，发现其主要作用靶点是调节铁代谢及脂质过氧化相关信号通路，进而抑制肿瘤的生长、增殖、转移并逆转耐药（表2^[75⇓⇓⇓⇓⇓⇓⇓⇓⇓⇓⇓⇓⇓⇓⇓⇓⇓⇓⇓⇓⇓⇓⇓⇓⇓⇓⇓⇓⇓-105]^、3^[106⇓⇓⇓⇓-111]^），且这些中药多具有清热解毒、行气活血、化痰软坚等功效。

**表2:** 中药活性成分调控NSCLC铁死亡的作用机制

Classification	Active ingredient	Primary origin	Model		IC_50_	Mechanism	Ref.
			In vitro	In vivo			
Terpenoids	Artesunate, Dihydroartemisinin	Artemisia annua	H1299 A549 LTEP-a-2 NCI-H23 NCI-H358	-	-	Inhibiting system Xc^-^, increasing mRNA levels of TFRC	^[75]^
	Dihydroartemisinin	Artemisia annua	A549 A549-GR H1975 HCC827	-	-	Increasing ROS	^[76]^
	Andrographolide	Andrographis paniculata	H460 H165	C57 mice	33.16 32.45 (μmol/L)	Increasing intracellular Fe^2+ ^and lipid ROS, reducing GSH content, inhibiting GPX4 and SLC7A11 expression	^[77]^
	Celastrol	Tripterygium wilfordii	HCC827 A549 H1299	BALB/c nude mice	-	Increasing ROS generation, disrupting MMP	^[78]^
	Manoalide	Sponge	A549 H157 HCC827 PC9	C57 mice BALB/c nude mice	-	Inhibiting Nrf2-SLC7A11 axis, activating FTH1 pathway by mitochondrial Ca^2+ ^overloading	^[79]^
	Oridonin	Rabdosia rubescens	A549 A549/DDP	-	-	Inducing LPO and Fe^2+ ^overload, inhibiting the expression of system Xc^-^, GPX4, FTH1, Nrf2	^[80]^
β-elemene								Radix curcumae	A549 NCI-H460 SPC-A-1	NOD/SCID Mice	-	Activating TFEB gene, upregulating transcription levels of downstream genes GLA, MCOLN1, and SLC26A11, increasing ubiquitination and degradation of GPX4	^[81]^
α-hederin	Ivy vine	A549 PC9	BALB/c nude mice	14.94 13.93 （μg/mL）	Disruption of GSS/GSH/GPX2 axis mediated GSH redox system	^[82]^
Dihydroisotanshinone I	Salvia miltiorrhiza	A549 H460	BALB/c nude mice	-	Blocking the protein expression of GPX4	^[83]^
Betulin	Betula platyphylla	A549 H460	BALB/c nude mice	-	Increasing the expression of ROS, Fe^2+^, MDA, and inhibiting the expression of GSH	^[84]^
Sclareol	Salvia sclarea	A549	-		Downregulation of FPN and GPX4 expression	^[85]^
Saponins	Timosaponin AIII	Anemarrhenae rhizoma	H1299 A549	C57 mice BALB/c nude mice	3.04 2.57 (μmol/L)	Increasing ROS, Fe^2+^, MDA, and ubiquitination and degradation of GPX4, inhibiting GSH, and targeting the degradation of GPX4 by binding to HSP90	^[86]^
	Ophiopogonin-B	Ophiopogon japonicus	A549	BALB/c nude mice	-	Activating AURKA, PHKG2, inhibiting SLC7A5, increasing intracellular Fe^2+^, MDA, reduce GSH, disrupting MMP	^[87]^
	Brusatol	Brucea javanica	A549 NCI-H838 NCI-H1703 BEAS-2B	NOG mice	-	Activating the Nrf2/FOCAD-FAK signaling pathway, depriving cysteine	^[88]^
Alkaloid	Solasonine	Solanum nigrum	Calu-1 A549	-	-	Increasing Fe^2+ ^and ROS levels, inhibiting GSH, GPX4, SLC7A11 expression, inducing mitochondrial oxidative stress and lipid peroxidation	^[89]^
	Sinapine	Sinapis alba	A549 SK H661	BALB/c nude mice	-	Increasing intracellular Fe^2+^, lipid peroxidation, and ROS, inhibiting P53/SLC7A11 pathway	^[90]^
	Sanguinarine	Macleaya cordata	A549 H3122	BALB/c nude mice	-	Increasing Fe^2+ ^, ROS, MDA content, reducing GSH content, and endogenous GPX4 ubiquitination mediated by E3 ligase STUB1	^[91]^
	Fascaplysin	Sponge	A549	C57 mice	2.923 (μmol/L)	Increasing ROS and Fe^2+ ^levels, downregulate ferroptosis-related proteins and endoplasmic reticulum stress	^[92]^
	Camptothecin	Camptotheca acuminata	A549 LLC	BALB/c nude mice	268.2 1278 (nmol/L)	Increasing intracellular ROS and LPO, reducing GSH and GPX4	^[93]^
	Capsaicin	Chili pepper	A549, NCI-H23	-	-	Inhibiting SLC7A11/GPX4 signaling pathway	^[94]^
	Cinobufotalin	Venenum bufonis	A549 SK-MES-1	-	-	Activating lncRNA LINC00597/hsa miR-367-3p/TFRC pathway	^[95]^
Flavone	6-Gingerol	Ginger	A549	BALB/c nude mice	-	Downregulating USP14 expression, increasing ROS and Fe^2+^ concentrations, increasing the number of autophagosomes	^[96]^
	Curcumin	Curcuma longa	A549 H1299	C57 mice	-	Increasing Fe^2+^, ACSL4 protein levels, consuming GSH, inhibiting SLC7A11 and GPX4 expression	^[97]^
	Curcumin analog HO-3867	Curcuma longa	A549 H460 PC9 H1975 H1299 A549 p53 KO H460 p53 KO	-	37.6 23.7 42.1 28.7 >200 >200 >200 (μmol/L)	Activating P53-DMT1 axis, inhibiting GPX4	^[98]^
	Ginkgetin	Gingko biloba	A549 NCIH460 SPC-A-1	BALB/c nude mice	-	Inhibiting Nrf2/HO-1 axis, SLC7A11, GPX4 expression, GSH/GSSG ratio, increasing ROS	^[99]^
	Eriocitrin	Citrus limon	A549 H1299	-	-	Increasing Fe^2+^ and ROS levels, inhibiting the expression of Nrf2, SLC7A11 and GPX4	^[100]^
	2-methoxy-6-acetyl-7-methyljuglone	Polygonum cuspidatum	A549CR H1975AR	Zebrafish	7.5 2.5 (μmol/L)	Activating and binding NQO1, increasing ROS, LIP and LPO	^[101]^
Others	Erianin	Dendrobium candidum	H460 H1299	BALB/c nude mice	-	Increasing ROS accumulation, LPO, and GSH depletion through the Ca^2+^/CaM pathway	^[102]^
	Bufotalin	Toad crisp	A549	BALB/c nude mice	4.21±0.90 (μmol/L)	Increasing intracellular Fe^2+^, inhibiting GPX4 protein expression, accelerating GPX4 degradation and ubiquitination	^[103]^
	Red ginseng polysaccharide	Red ginseng	A549	-	376.2 (μmol/L)	Increasing LDH, inhibiting GPX4 expression	^[104]^
	Scoparone	Artemisia scoparia	A549 H1299 PC-9	-	-	Activating ROS/JNK/SP1/ACSL4 axis	^[105]^

IC_50_: half maximal inhibitory concentration; DDP: Cisplatin; TFEB: transcription factor EB; MCOLN1: mucolipin 1; SLC26A11: solute carrier family 26 member 11; GSS: glutathione synthetase; GPX2: glutathione peroxidases 2; HSP90: heat shock protein 90; AURKA: aurora kinase A; PHKG2: phosphorylating enzyme kinase G2; SLC7A5: solute carrier family 7 member 5; USP14: ubiquitin carboxyl-terminal hydrolase 14; LIP: lipase inhibitory protein; JNK: c-Jun N-terminal kinase; SP1: specifieity protein 1.

**表3:** 单味中药和中药复方调控NSCLC铁死亡的作用机制

Single-herb traditional Chinese medicine/Chinese herbal compounds	Composition of Chinese medicine	Model		IC_50_	Mechanism	Ref.
		In vitro	In vivo			
Realgar	Realgar	H23 A549 H460 H1650	-	2.66±0.33 4.66±0.76 7.02±0.36 7.96±1.00 (μg/mL)	Inhibiting expression of GPX4, SCL7A11, increasing expression of ACSL4	^[106]^
Huaier ointment	Trametes robiniophila Murr	H1299	-	-	Increasing the content of ROS	^[107]^
Fuzheng Kang-Ai decoction	Radix pseudostellariae, Atractylodes rhizoma, Astragalus，etc.	A549 H1299 PC9 H1650	BALB/c nude mice	-	Inhibiting GPX4 protein and mRNA levels, SLC7A11 and SLC3A2, reducting GSH/GSSG ratio	^[108]^
Qingrehuoxue formula	Scutellaria baicalensis, red peony paeoniae, etc.	-	BALB/c nude mice	-	Activating P53 and inhibiting GSK-3 β/Nrf2 signaling pathway, increaseing ROS, Fe^2+^, H_2_O_2_, and MDA levels, inhibiting GSH, GPX4, and SLC7A11 protein levels	^[109]^
Xiaoyi Sanjie formula	Rehmannia, Toosendania, Trichosanthes, Coix seed, etc.	A549 PC-9	C57 mice	5.814 5.115 (mg/mL)	Increasing ROS levels, inhibiting GPX4 and FTH1 protein expression	^[110]^
Sijunzi decoction	Ginseng, Atractylodes rhizoma, Poria cocos	A549 A549/DDP	BALB/c nude mice	-	Inhibiting the P62/KEAP1/Nrf2 pathway, increasing lipid peroxidation and autophagy	^[111]^

GSK-3β: glycogen synthase kinase-3 beta; GSSG: glutathione disulfide; P62: sequestosome-1.

### 2.1 中药活性成分

#### 2.1.1 萜类（Terpenoids）

萜类化合物是一类广泛存在于植物中的天然有机化合物，它们由多个异戊二烯单元组成，部分萜类化合物有抗菌、抗炎、抗肿瘤作用^[112]^。青蒿琥酯与双氢青蒿素（Artesunate, Dihydroartemisinin）是青蒿素衍生物，它们可以下调System Xc^-^并上调转铁蛋白受体（transferrin receptor, TFRC）的mRNA水平诱导铁死亡，另外双氢青蒿素还能通过铁死亡逆转NSCLC细胞对吉非替尼耐药^[75,76]^。穿心莲内酯（Andrographolide）能提高细胞内Fe^2+^和ROS含量，抑制GPX4和SLC7A11的表达导致铁死亡，还会导致MMP去极化和线粒体ATP合成减少，并破坏线粒体功能^[77]^。雷公藤红素（Celastrol）与Erastin联用时可以增加ROS生成，破坏MMP，促进线粒体分裂导致铁死亡，并以热休克转录因子1（heat shock transcription factor 1, HSF1）依赖的方式启动ATG5/ATG7依赖的自噬，PINK1/Parkin（PTEN-induced putative kinase 1/parkin RBR E3 ubiquitin protein ligase）依赖的线粒体自噬^[78]^。马诺内酯（Manoalide）通过抑制Nrf2-SLC7A11轴和线粒体Ca^2+^超载诱导的FTH1通路导致铁死亡，并通过产生ROS抑制KRAS-ERK通路，进而逆转KRAS突变NSCLC细胞对奥希替尼耐药性^[79]^。冬凌草甲素（Oridonin）能诱导LPO和Fe^2+^过载，抑制System Xc^-^、GPX4和FTH1、Nrf2的表达诱导铁死亡，并上调自噬代表蛋白LC3B的表达诱导自噬，通过铁死亡和自噬逆转A549细胞顺铂耐药^[80]^。β-榄香烯（β-elemene）显著激活转录因子EB（transcription factor EB, TFEB）并上调下游基因GLA、MCOLN1、SLC26A11的转录水平，促进GPX4的泛素化和降解，诱导细胞铁死亡^[81]^。α-常春藤素（α-hederin）通过破坏GSS/GSH/GPX2轴介导的GSH氧化还原系统来诱导NSCLC的铁死亡，并增强顺铂化疗的敏感性^[82]^。二氢异丹参酮I（dihydroisotanshinone I）可以阻断GPX4的蛋白表达诱导铁死亡，在体内通过铁死亡和凋亡抑制NSCLC肿瘤的生长^[83]^。白桦皮甙（Betulin）与吉非替尼联用时通过铁死亡来逆转表皮生长因子受体（epidermal growth factor receptor, EGFR）野生型/Kirsten大鼠肉瘤病毒癌基因同源物（kirsten rat sarcoma viral oncogene, KRAS）突变NSCLC细胞吉非替尼耐药，其具体机制为增加细胞内ROS、Fe^2+^和MDA的含量，减少GSH表达^[84]^。香紫苏醇（Sclareol）可以抑制FPN和GPX4的表达诱导铁死亡，并能上调PARP1[poly (ADP-ribose) Polymerase 1]和pro-Caspase 3/9的表达，下调分裂和抗凋亡蛋白Bcl-2和Bcl-xL的表达促进NSCLC细胞凋亡^[85]^。

#### 2.1.2 皂苷类（Saponins）

皂苷是一类广泛存在于植物中的天然糖苷，分为三萜类和甾体类皂苷，具有多种生物活性，包括抗肿瘤、抗炎、免疫调节等作用^[113]^。知母皂苷AIII（Timosaponin AIII）通过诱导ROS的释放和Fe^2+^蓄积、MDA的产生和GSH减少以及GPX4泛素化和降解导致铁死亡，并可以与热休克蛋白90（heat shock protein 90, HSP90）结合形成复合物，进一步靶向降解GPX4^[86]^。麦冬皂苷B（Ophiopogonin B）通过上调AURKA（aurora kinase A）和PHKG2，下调SLC7A5（solute carrier family 7 member 5）表达，导致细胞内MDA和Fe^2+^升高、GSH降低和MMP紊乱诱导铁死亡^[87]^。Nrf2抑制剂鸦胆子苦醇（Brusatol）通过抑制Nrf2活性，激活FOCAD-FAK（focadhesin-focal adhesion kinase）通路导致NSCLC细胞内半胱氨酸的剥夺诱导铁死亡^[88]^。

#### 2.1.3 生物碱类（Alkaloid）

生物碱由一个或多个芳香族或脂肪族环组成，其中至少一个环含有氮原子，通常是吡啶或喹啉结构，部分生物碱具有抗肿瘤、抗菌、调节生理功能等作用^[114]^。龙葵碱（Solasonine）诱导脂质过氧化，上调Fe^2+^和ROS的水平，抑制GSH、GPX4、SLC7A11等的表达和诱导线粒体氧化应激导致铁死亡^[89]^。芥子碱（Sinapine）可以增加脂质过氧化和ROS，并通过抑制P53/SLC7A11通路诱导铁死亡，同时上调TF与TfR1的活性导致细胞内Fe^2+^含量增多^[90]^。血根碱（Sanguinarine）可以增加Fe^2+^浓度、ROS水平和MDA含量以及降低GSH含量，并且通过E3连接酶STUB1（STIP1 homology and U-box containing protein 1）介导的内源性GPX4的泛素化从而降低GPX4的蛋白稳定性，进而诱导铁死亡^[91]^。海洋生物活性碱（Fascaplysin）可以A549细胞内ROS和Fe^2+^水平，下调铁死亡相关蛋白和内质网应激诱导铁死亡，通过调节Wnt/β-catenin信号通路和逆转上皮间充质转化表型来抑制迁移，并且还能显著上调肺癌细胞中PD-L1的表达进而增强免疫治疗效果^[92]^。载有喜树碱（Camptothecin）的中药纳米粒能导致A549细胞ROS、LPO产生增多，GSH、GPX4减少引起铁死亡，通过铁死亡和凋亡途径增强光热疗法（photothermal therapy, PTT）疗效^[93]^。辣椒碱（Capsaicin）通过失活SLC7A11/GPX4信号通路诱导铁死亡，抑制NSCLC细胞增殖^[94]^。华蟾素（Cinobufotalin）通过lncRNA LINC00597/hsa-miR-367-3p/TFRC通路诱导NSCLC铁死亡^[95]^。

#### 2.1.4 黄酮类（Flavonoids）

黄酮类化合物是许多中药的有效成分，其具有多种生物活性，包括抗癌、抗炎、抗菌等作用^[115]^。6-姜酚（6-Gingerol）可以抑制USP14（ubiquitin-specific protease 14）的表达并调控自噬依赖性铁死亡来抑制NSCLC肿瘤增长^[96]^。姜黄素（Curcumin）能够激活自噬，增加自噬蛋白Beclin1和LC3的表达，诱导铁过载，脂质过氧化，GSH耗竭，升高ACSL4蛋白水平，降低SLC7A11和GPX4蛋白表达等，并通过自噬途径诱导NSCLC细胞发生铁死亡^[97]^。姜黄素类似物HO-3867（Curcumin analog HO-3867）通过激活P53-DMT1轴和抑制GPX4诱导NSCLC发生铁死亡，并且能通过抑制Mcl-1和Bcl-2表达、上调Bax表达诱导细胞凋亡^[98]^。银杏素（Ginkgetin）通过抑制Nrf2/HO-1轴并下调SLC7A11和GPX4的表达和GSH/GSSG比值，促进ROS生成诱导铁死亡，并且能够增强NSCLC细胞顺铂敏感性^[99]^。圣草次苷（Eriocitrin）增加NSCLC细胞的ROS水平，下调Nrf2、SLC7A11和GPX4的表达诱导铁死亡，并通过铁死亡途径抑制上皮间充质转化^[100]^。2-甲氧基-6-乙酰基-7-甲基胡桃酮（2-methoxy-6-acetyl-7-methyljuglone）可以激活并结合NQO1，引发ROS生成、LIP（lipase inhibitory protein）增加和脂质过氧化诱导细胞铁死亡，并逆转A549细胞顺铂耐药和H596细胞奥西替尼耐药^[101]^。

#### 2.1.5 其他

毛兰素（Erianin）是从石斛中分离得到的联苄类化合物，它通过Ca^2+^/CaM依赖性的通路诱导铁死亡的发生，并能抑制细胞迁移发挥抗癌作用^[102]^。蟾毒它灵（Bufotalin）是从蟾酥中提取的类固醇内酯类化合物，它增加了细胞内的Fe^2+^和GPX4的泛素化，显著抑制A549细胞增殖并诱导其发生铁死亡^[103]^。红参多糖（Red ginseng polysaccharide）属于多糖类化合物，它是从红参中提取得到的一种主要活性成分，能诱导A549细胞乳酸脱氢酶（lactate dehydrogenase, LDH）释放，抑制GPX4表达诱导铁死亡^[104]^。滨蒿内酯（Scoparone）是从滨蒿中提取的苯丙素类化合物，其可以激活ROS/JNK/SP1/ACSL4轴触发NSCLC细胞铁死亡^[105]^。

### 2.2 单味中药和复方

雄黄（Realgar）通过提高细胞ACSL4、Fe^2+^、ROS，降低GPX4、SCL7A11、GSH的水平引发NSCLC铁死亡，索拉芬尼可以促进雄黄诱导的铁死亡^[106]^。欧海亚等^[116]^通过网络药理学和分子对接发现35种可以调控铁死亡的中药化合物，将35种化合物在TCMSP数据库中匹配得到301味中药，这些中药药味以苦、辛为主，药性以寒居多，主要归肝、肺二经。其中包括白果、枇杷叶、葛花、余甘子、麻黄根、槐角、女贞子和沙棘。这些中药成分可能与多个靶点有关联，因此推测它们可能对铁死亡具有一定的调节作用。但在NSCLC铁死亡的具体机制还需进一步探讨，因此对于中药干预铁死亡的潜力亟待挖掘。

槐耳清膏（Huaier ointment）是一种传统中药制剂，可以增加ROS的含量诱导铁死亡并抑制H1299细胞NSCLC生长和转移^[107]^。扶正抗癌汤（Fuzheng Kang-Ai decoction）通过抑制GPX4的蛋白和mRNA水平，抑制SLC7A11和SLC3A2，并且降低GSH/GSSG比率诱导NSCLC铁死亡，而过表达GPX4显著逆转扶正抗癌汤诱导铁死亡的效果^[108]^。清热活血方（Qingrehuoxue formula）激活P53和抑制GSK-3β/Nrf2信号通路显著增加ROS、Fe^2+^、H_2_O_2_和MDA的水平，抑制GSH、GPX4和SLC7A11的蛋白水平引发NSCLC铁死亡^[109]^。消异散结方（Xiaoyi Sanjie formula）上调ROS水平，抑制GPX4和FTH1的蛋白表达诱导A549和PC9细胞铁死亡，其中白杨素、芹菜素和山柰酚可能是诱导铁死亡的关键成分^[110]^。四君子汤（Sijunzi decoction）因其四味中药药力平和，补益作用如君子而得名，常用于肺癌晚期患者。有研究^[111]^表明该方通过抑制P62/Keap1/Nrf2通路诱导脂质过氧化和自噬依赖性的铁死亡，并提高NSCLC细胞的顺铂敏感性，这为四君子汤的应用提供了新思路。通过汇总发现具有活血化瘀、清热解毒、补气生血等功效的中药复方在诱导铁死亡治疗NSCLC中发挥重要作用。

## 3 总结与展望

铁死亡是一种铁依赖的、ROS异常累积导致膜质过氧化而引起的一种新型的PCD方式，其作为一种新的细胞死亡方式，在抗肿瘤治疗方面也发挥着重要作用。在NSCLC中，诱导铁死亡可以抑制肿瘤细胞生长、增殖、上皮间充质转换，甚至可以逆转顺铂、吉非替尼、帕博利珠单抗等药物耐药性^[117⇓-119]^。随着对铁死亡的研究不断深入，越来越多的调控机制被不断发现，目前研究多针对System Xc^-^/GPX4通路，而对FSP1/CoQ10、DHODH/CoQ10、Nrf2、P53等通路的研究尚不深入。另外，一些研究^[120,121]^认为铁死亡是依赖自噬的一种死亡途径，但关于自噬具体如何调控铁死亡的机制尚不完全明确。因此寻找新的铁死亡调控靶点并阐明这些靶点在NSCLC中的作用迫在眉睫。

中药是我国宝贵的资源，在治疗许多疾病方面有着悠久的历史，其成分复杂、作用靶点多，在治疗NSCLC方面也有独特的优势。中药在调控铁死亡方面也发挥着多靶点的优势，一些中药活性成分和复方可以作用于各种铁死亡相关信号通路或调节蛋白，促进NSCLC细胞铁死亡发挥抑制肿瘤生长、增殖、转移、上皮间充质转化，并逆转耐药性的作用。然而，中药调控铁死亡治疗NSCLC等疾病仍有许多问题需要解决，关于中药调控铁死亡的研究多集中在中药活性成分，但中药治疗疾病并不依赖于特定的活性成分，而往往涉及多种成分的共同作用，了解各个成分在疾病中发挥的作用，可以为中药拟方提供更多理论依据；中药纳米粒可以增强药物在肿瘤组织中的积累和铁死亡靶向性，提高抗肿瘤药物的疗效，并减少对健康组织的损伤，具有良好的应用前景，未来需要深入研究中药纳米粒子使其发挥更大的作用；目前关于中药干预铁死亡机制的研究主要集中在动物和细胞水平，高质量的临床研究相对缺乏，如何通过调控铁死亡指导中药防治NSCLC发挥其优势，还需要进一步探讨。
